# Structure/Function/Dynamics of Photosystem II Plastoquinone Binding Sites

**DOI:** 10.2174/1389203715666140327104802

**Published:** 2014-06

**Authors:** Maya D. Lambreva, Daniela Russo, Fabio Polticelli, Viviana Scognamiglio, Amina Antonacci, Veranika Zobnina, Gaetano Campi, Giuseppina Rea

**Affiliations:** 1 Institute of Crystallography, National Research Council, Monterotondo, Italy;; 2 Istituto Officina dei Materiali, National Research Council, c/o Institut Laue-Langevin, Grenoble, France, and Institut Lumière Matière, Université de Lyon 1, France;; 3 Department of Sciences, University Roma Tre, Rome, Italy;; 4 National Institute of Nuclear Physics, Roma Tre Section, Rome, Italy

**Keywords:** Molecular docking, molecular dynamics simulations, Photosystem II, plastoquinone, plastoquinone binding site, protein dynamics.

## Abstract

Photosystem II (PSII) continuously attracts the attention of researchers aiming to unravel the riddle of its functioning and efficiency fundamental for all life on Earth. Besides, an increasing number of biotechnological applications have been envisaged exploiting and mimicking the unique properties of this macromolecular pigment-protein complex. The PSII organization and working principles have inspired the design of electrochemical water splitting schemes and charge separating triads in energy storage systems as well as biochips and sensors for environmental, agricultural and industrial screening of toxic compounds. An intriguing opportunity is the development of sensor devices, exploiting native or manipulated PSII complexes or *ad hoc* synthesized polypeptides mimicking the PSII reaction centre proteins as bio-sensing elements. This review offers a concise overview of the recent improvements in the understanding of structure and function of PSII donor side, with focus on the interactions of the plastoquinone cofactors with the surrounding environment and operational features. Furthermore, studies focused on photosynthetic proteins structure/function/dynamics and computational analyses aimed at rational design of high-quality bio-recognition elements in biosensor devices are discussed.

## 1. STRUCTURAL OVERVIEW OF PSII CORE COMPLEX

Photosynthetic organisms have the unique ability of entrapping and using solar energy to oxidize water to molecular oxygen through the highly structured multi-subunit protein-chlorophyll complexes of photosystem I (PSI) and photosystem II (PSII), arranged in supra-molecular assemblies embedded in thylakoid membranes. These structured assemblies are organized in a way to ensure supreme energy conversion efficiency and effective diffusion of small molecules (plastoquinone) interconnecting the two photosystems functions in a fine-tuned electron transport chain and leading to the synthesis of ATP and reducing equivalents. PSII stands out for its remarkable ability to act as a water:plastoquinone oxidoreductase, extracting electrons from water in physiological conditions. The outstanding features of PSII have motivated numerous research efforts aimed to clarify its structural and functional relationships, which until recently have been hindered by the lack of a high resolution X-ray crystal structure. Our current understanding of the PSII structure/function/dynamics rationale has been largely acquired by comparative studies and assumptions with the simpler and better-known purple bacterial reaction centre (bRC) [for review on bRC see 1,^ 2^]. In the last decade, the crystal structure resolution at 2.9-3.5 Å has exponentially improved the knowledge of atomic details of dimeric and monomeric forms of PSII core complex (PSIIcc) of the thermophilic cyanobacterium *Thermosynechococcus elongatus* [3-7]. More recently, Umena and coworkers provided the highly resolved 1.9 Å structure from *Thermosynechococcus vulcanus* [8]. This structure features a rich collection of atomic details among which a cofactors arrangement made up by 35 chlorophylls, 2 pheophytins, 11 β-carotenes, 2 plastoquinones, 2 heme irons, 1 non-heme iron, 4 Mn atoms, 3-4 Ca atoms (among which, 1 is in the Mn_4_Ca-cluster), 3 Cl^-^ ions (among which, 2 are in the vicinity of the Mn4Ca-cluster), and 1 (bi)carbonate ion. From the electron density map it was possible for the first time to locate all the atoms building up the Mn_4_CaO_5_-cluster and to define the precise position of all the metal ligands and of the substrate water molecules. In this high-resolution structure the primary plastoquinones (PQs) Q_A_ and Q_B_ occupy positions very similar to those observed in previous crystallographic studies. However, the third plastoquinone molecule observed in the structure solved by Guskov and coworkers [5], the so called Q_C_, has not been observed in this last structure casting some doubts on the real physiological function of Q_C_. These structural data together with the numerous biochemical and spectroscopic methods, and computational analyses contribute to solve the enigma of PSII structure/function/dynamics specific correlation. 

**Fig. (1):**
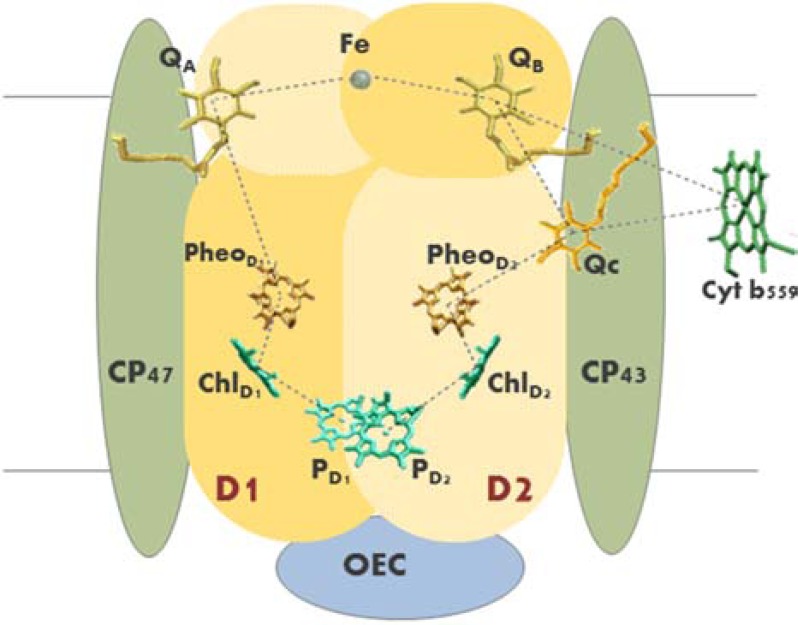
Schematic representation of PSIIcc main protein components and electron transfer cofactors. The reaction centre (D1/D2) heterodimer is reported in light (D2) and dark (D1) yellow, respectively; core antenna proteins CP47 and CP43 are in green; the Oxygen Evolving Complex (OEC) – in blue. PSIIcc associated cofactors: the primary (PD1/PD2) and accessory (ChlD1/ChlD2) Chl*a* molecules are in cyan, pheophytin a (PheoD1/PheoD2) are in gold brown, plastoquinones (Q_A_, Q_B_ and Q_C_) are in yellow, the heme of cytochrome *b_559_* (Cyt. *b_559_*) is colored green and the non-heme iron in grey.

PSIIcc consists about 20 different polypeptide chains, with the majority in the form of trans-membrane and *α*-helical subunits. Among these, PsbA (D1) and PsbD (D2) subunits, with five trans-membrane helices (TMH) each, form the architecture of PSII RC and host the major redox-active cofactors organized in two quasi symmetrical branches: two main chlorophyll *a* (Chl*a*), PD1 and PD2, two accessory Chl*a*, ChlD1 and ChlD2, two peripheric Chl*a*, ChlzD1 and ChlzD2, two Pheophytin *a*, PheoD1 and PheoD2, and two PQs, Q_A_ and Q_B_ (Fig. **1**). On the stromal (cytosol in prokaryotes) side of the complex between the primary and secondary PQs, a non-heme iron interacting with a bicarbonate anion has been also revealed. It is electrostatically associated to the two PQs, optimizing charge distribution and H-bonding of the Q_A_-Fe-Q_B_ bridge and contributing to the stability of PQs when reduced to semiquinones (SQ) [9, 10]. Two antenna proteins, PsbB (CP47) and PsbC (CP43), are located in close proximity of the D1/D2 heterodimer. Their structure consists of six TMHs each and large extrinsic loops at the lumenal side. They host the majority of PSIIcc chlorophyll molecules (16 and 13 Chl*a*, respectively) and, besides light harvesting, are involved in the assembly and stabilization of the Oxygen Evolving Complex (OEC) active conformation [8, 11]. Although the resolution of the PSII crystal structure at 1.9 Å provided an improved picture of the overall configuration of the OEC Mn_4_CaO_5_ cluster, and atomic distances of the Mn-Mn and Mn-Ca interaction network, the structural changes occurring during catalysis are still under investigation. Different X-ray absorption spectroscopy techniques and quantum mechanical/molecular mechanics calculations are providing further insights into the relative position of one of the bridging oxygen in the Mn_4_CaO_5_ cluster, that are crucial for a detailed understanding of the O-O bond formation mechanism during the water oxidation reaction [12, 13]. An overview of the kinetics and thermodynamics of water oxidation and new knowledge on biogenesis and assembly of OEC has been critically addressed recently by Vinyard and coworkers [14]. Among the major intrinsic proteins necessary for PSII function are the TMHs of PsbE and PsbF, corresponding to *α-* and *β-*subunit of the heme-containing protein cytochrome *b_559_* (Cyt.*b_559_*), which most probably takes an active part in PSII photoprotection mechanisms. However, its involvement in PSII forward electron transport seems unlikely, despite its location close to the secondary quinone binding site [15, 16].

Important molecular players of PSIIcc are also carotenoid (Car) molecules; the position of 12 β-carotenes per PSIIcc monomer has been assigned in the PSII structure refined at 2.9 Ǻ resolution [5]. It has been proposed that these poly-isoprenoid compounds can play crucial roles in light-harvesting, excitation energy transfer regulation, Chl triplet states quenching, singlet oxygen scavenging, and structure stabilization [17]. Crystallographic studies have also revealed up to 25 integral lipids associated to each PSIIcc monomer, including 11 monogalactosyldiacylglycerol (MGDG), 7 digalactosyldiacylglycerol (DGDG), 5 sulfoquinovosyl-diacylglycerol (SQDG), and 2 phosphatidylglycerol (PG), having almost full compositional and spatial overlap in PSII monomeric and dimeric form [5, 7, 8]. PSII lipid content and composition denote the typical thylakoid membrane distribution and are characterized by spatial heterogeneity: the head group has a stromal (cytoplasmic in prokaryote) orientation for the negatively charged PG and SQDG, lumenal for DGDG and bilateral for MGDG. PSII integral lipids behave more as a dynamic multifunctional cofactor into overall functioning of the complex than as a simply hydrophobic surrounding matrix. Lipid molecules, in fact, actively interact with the Mn-cluster components and stabilize its assembly; contribute to a different extent to define the configuration of approximately 1/3 of Chl*a* and 3/4 of Car molecules in PSII, and last but not least, largely contribute to the PSII monomer-monomer interactions and complex dimerization [18, 19]. Integral lipids of PSIIcc play a special role also in defining the large internal cavity for the PQ/PQH_2_ exchange, representing the ideal flexible scaffold to host the hydrophobic PQ isoprenoid residues, including the recently identified PQ molecule, Q_C_. The cavity has two openings directed towards the membrane heart, approximately in the centre of the membrane thickness, defined as channel I and channel II [5]. Channel I is bigger than channel II, lodges Q_C_, and opens between TMHs of PsbJ protein and Cyt.*b_559_*, while the recently described channel II accommodates Q_B_ phytyl tail and is formed by TMH-*d* and *e* of D1, TMH-*a* of D2 and TMH of PsbF (*β*-subunit of Cyt.*b_559_*). 

## 2. PSII PLASTOQUINONES

The two chemically identical PQ molecules, hosted into the Q_A_ and Q_B_ binding sites of the RC heterodimer, have the distinctive role of stabilization of primary charge separation and conduction of electrons towards the photosynthetic electron transport chain, respectively. The existence of a third plastoquinone, Q_C_, in the PSII core complex has been supported by Cyt.*b*_559_ photoreduction and redox titration experiments and lately identified by the 2.9 Å resolution crystallographic study [5, 20-22]. The role of this quinone molecule in the PSII function is still under debate and will be discussed below. The binding energies for Q_A_, Q_B_ and Q_C_ obtained by fragment molecular orbital (FMO) calculations based on the 2.9 Å X-ray structure are -56.1 kcal/mol, -37.9 kcal/mol and -30.1 kcal/mol, respectively [23]. The FMO calculations indicated a decrease of the relative contribution of the PQ/protein matrix interactions to the total PQ interaction energy in the order Q_A_>Q_B_>Q_C_ with concomitant increase of the relative contribution of surrounding lipids and cofactors (Table **1**). Thus, the FMO results drew a correlation between the increasing of PQ mobility and the growing relative contribution of lipids and cofactors interaction.

### 2.1. Plastoquinone at Q_A_ Binding Site

**Fig. (2):**
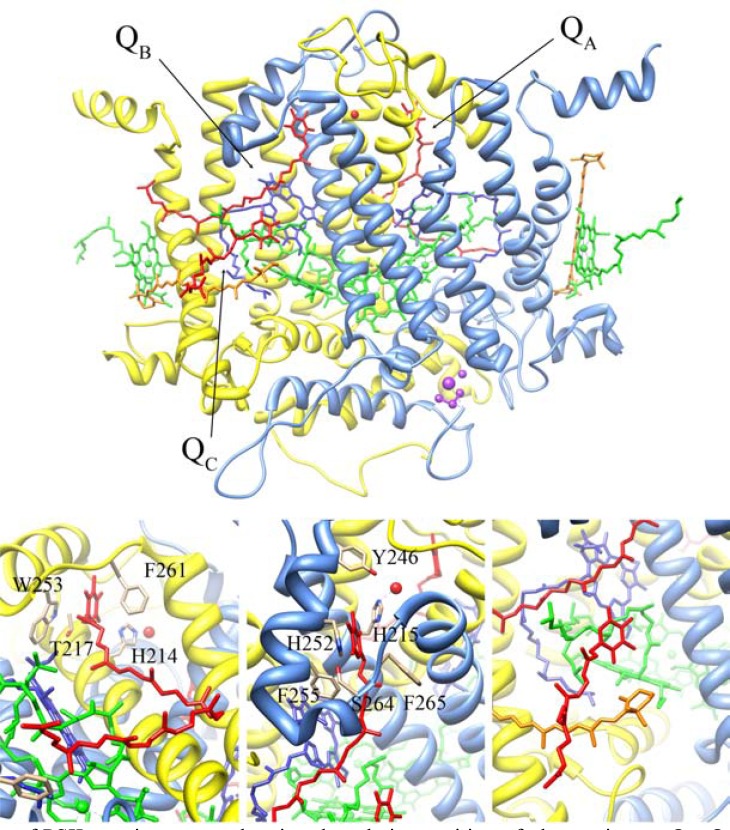
**Top:** Schematic view of PSII reaction centre showing the relative position of plastoquinones Q_A_, Q_B_ and Q_C_ as red sticks. Chlorophylls, pheophytins and carotenoids are also shown (in green, blue and orange sticks, respectively). The OEC Mn_4_Ca cluster and the non-heme iron atom are represented by purple and red spheres, respectively. D1 and D2 backbone are shown as a blue and yellow ribbon, respectively. **Bottom:** Detailed view of the plastoquinones Q_A_ (left panel), Q_B_ (centre panel), and Q_C_ (right panel) binding sites. Residues mentioned in the text are shown as sticks coloured by atom type.

The plastoquinone at Q_A_ site tightly interacts with the RC protein matrix, does not undergo protonation, and is not exchangeable. It functions as a single electron recipient and mediates the double reduction of the second quinone molecule at the Q_B_ binding pocket. In contrast, the double reduced and protonated Q_B_ moves away from its binding site and is promptly replaced by a new PQ molecule (as well as by artificial quinones or herbicides), leading to consider this molecule more as a substrate rather than a tightly bound cofactor. Although chemically identical, Q_A_ and Q_B_ demonstrate distinguishable redox potentials, necessary for the potentiation of PSII forward electron transfer and the fine tuning of the backward recombination reactions [24, 25]. The different functional and redox-properties of the PSII plastoquinone molecules are largely determined by the interactions with the surrounding environment (H-bonding, hydrophobic interfaces, π-stacking), and the conformation of the individual binding sites and phytyl chains [4-6]. FMO quantum chemical calculations based on the 2.9 Å X-ray structure of *T. elongatus* pointed out that the Q_A_ phytyl tail is responsible for more than 60% of the total molecule interactions and Q_A_ stabilization is mainly due to interactions with amino acid residues [23], Table **1** and Table **2**. The position of the isoprenoid tail is restrained mostly by van der Waals contacts with TMH-*d* of D2, TMH-*a* of D1, PsbL and PsbT and the accessory ChlD1 (Fig. **2**). The 2.9 Å resolution structure revealed an additional stabilization of the Q_A_ molecule due to the interaction of the terminal unit of the isoprenoid tail with π-electron clouds of the phenyl rings of D1-Phe52 and PsbT-Phe10 [5]. Q_A_ head is fixed by two H-bonds, formed by the keto-oxygen atoms with the D2-Phe261 backbone amide group and the side chain N of D2-His214, and by π-stacking with D2-Trp253 indole ring, occurring in an offset-stacked manner [4, 5]. The static picture of Q_A_ interactions within its binding niche obtained by X-ray analyses has been further complemented by spectroscopic studies. Simultaneous ^1^H and ^14^N Two-Dimensional Hyperfine Sublevel Correlation spectroscopy shed new light on the strength and orientation asymmetry of H-bonding of Q_A_ anion radical, establishing that when Q_A_ is in its reduced form, the H-bond involving D2-Phe261 amide group is significantly stronger than the one attributed to D2-His214 [26, 27]. Similarly, computational studies provided stronger interaction energies of Q_A_ with D2-Phe261, D2-Thr217 and D2-Trp253 than with Q_A_-D2-His214 [23], (Fig. **2**, left bottom panel).

Important properties of the primary PQ molecule, proven in bacteria, cyanobacteria and higher plants, is the existence of the one-electron redox couple Q_A_/Q_A_^•−^ in two conformations with highly different redox potential [28]. The well-recognized ability of herbicides binding at Q_B_ site to increase or decrease the susceptibility of PSII to photodamage was related to herbicide-induced changes at midpoint potential of the Q_A_ [29-31]. Recent studies have strengthened a previously found correlation [32, 33] between the Q_A_/Q_A_^•−^ midpoint potential changes and PSII donor side integrity level [28, 34]. The Q_A_ low potential form is present in functional PSII reaction centres while donor-side Ca^2+^ extraction or Mn cluster disassembly is associated with an increase (of approx. 150 mV) in the Q_A_ redox potential. Müh and coworkers [35] and Cardona and coworkers [36] have recently addressed in comprehensive reviews the physiological significance of the Q_A_ redox shift in guiding a “safe” PSII charge recombination reactions and protecting PSII from photodamage during the assembly of the RC. In addition, an up-shift of the Q_A_ midpoint potential by ca. +38 mV was observed in *T. elongatus* in photo-oxidative stress conditions, when in the PSII RC the PsbA3 (D1) isoform preferentially replaces the PsbA1 (D1) protein, expressed in optimal conditions. This finding evidenced the capability of the D1 amino acid composition to influence the Q_A_ redox potential, most probably affecting the Q_A_-His214(D2)-Fe-His215(D1)-Q_B_ interaction chain [37]. The rationale of the Q_A_ redox potential switching in photoprotection should be viewed in light of the very recent study conducted by Boussac and coworkers [38], demonstrating faster recombination reaction in PsbA3 than in PsbA1 PSII-RC, which could thwart ^1^O_2_ generation initiated by triplet state of the RC primary chlorophyll molecules.

### 2.2. Plastoquinone at Q_B_ Binding Site

The site hosting the secondary quinone, Q_B_, is situated in a symmetry-related manner to Q_A_ binding spot around the non-heme iron (Fig. **2**, top panel). Because of its substrate-like behaviour it was not always possible to assign the electron density found in the presumed Q_B_ binding site to a PQ molecule [6]. The problem of the Q_B_ binding site electron density inhomogeneity was surpassed by crystallization of the PSII core complex paired with a herbicide molecule [7]. However, in the 2.9 Å structure it was possible to model seven out of the nine Q_B_ isoprenoid units [5, 11]. In contrast to the secondary quinone acceptor in bRC, found in two differential positions called “proximal” and “distal” [39], PSII Q_B_ was located in only one position, corresponding to the “proximal” one in bRC. The Q_B_ binding site is formed mainly by the D1 RC protein residues (Table **2**). The keto-oxygen atoms of the Q_B_ head group form H-bonds with D1-Ser264 hydroxyl group and the side chain N of D1-His215. In contrast to Q_A_, Q_B_ has a possibility to form a third H-bond with the backbone amide group of D1-Phe265 (2.84 Å) [5, 8], (Fig. **2**, centre panel). Most probably, the latter is involved in the stabilization of the SQ state of the Q_B_ molecule during the reduction/protonation process [36]. The Q_B_ isoprenoid tail, located into the recently described PQ/PQH_2_ exchange channel II, is inclined in relation to the one of Q_A_ [5]. Similarly to Q_A_, the contribution of the phytyl tail to the total Q_B_ interaction energy is calculated to be more than 60%, but differently from the Q_A_ the surrounding protein matrix contributes for a smaller fraction of the binding energy [23], Table **1**. Based on the interaction strength of the Q_A_ and Q_B_ with the respective amino acid residues obtained by FMO calculations, Hasegawa and Noguchi [23] have questioned the role of the H-bonds established by the PQ keto-oxygen groups in the Q_A_ and Q_B_ binding. The authors observed more substantial contribution of the non-covalent interactions of Q_A_ head group with D2-Phe261 and D2-Trp253 and Q_B_ head group with D1-Phe255 and D1-Phe265 to the total PQ binding energies than the above mentioned H-bonding. Instead, it has been unambiguously demonstrated the importance of the H-bonding patterns of PSII PQs in determining their redox potential [40, 41]. The relative contribution of PSII integral lipids and cofactors to the total Q_B_ interaction energy is less than 20% and is mainly limited to the PQ tail (Table **1** and Table **2**). The Q_B_ phytyl chain is accommodated in channel II of the PSII internal cavity, connecting the Q_B_ binding site with the membrane PQ pool, and is in close interactions with the phytol chain ChlD2 and the acyl chains of MGDG18, and to a lesser extent to SQDG4, DGDG6 and MGDG7.

The reduction/protonation mechanism of the secondary quinone has been a long-term challenge for researchers in photosynthesis. The Q_B_ reduction occurs in two successive photoreactions including the formation of SQ intermediates. The first reaction is fast, occurring within 0.2-0.4 ms, and generates a SQ tightly bound into the Q_B_ pocket, which can accept a second electron in a somewhat slower process – 0.6-0.8 ms [42]. Experimental data suggested that Q_B_^•−^ becomes protonated before the second electron transfer occurs, thus avoiding the formation of highly charged and unstable Q_A_Q_B_^2−^ species [35, 36] and the protonation occurs more promptly if Q_A_ is in its reduced state [10].The resulting plastoquinol is characterized by low affinity for the Q_B_ binding niche and is promptly released into the membrane. 

In bRC the protonation process has been well established: the first H^+^ necessary to neutralize the charge on the distal (in relation to the non-heme Fe position) keto-oxygen atom of the SQ is provided by the Ser L223 amino acid trough Asp L213, while the second one is released by the Glu L212 residue. In plant and cyanobacterial PSII RCs the details of this phenomenon are still under investigation. Similarly to bRC, it has been supposed that the first protonation is accomplished by D1-Ser264 via D1-His252, while different possibilities have been explored for the mechanism of the second protonation [35, 36, 43]. Recently, by large-scale quantum mechanical/molecular mechanical approach based on the 1.9 Å resolution crystal structure, the D1-His215 was suggested as the sought H^+^ donor for the keto-oxygen of Q_B_H^−^ proximal to the non-heme Fe [10]. The authors speculated that the resulting D1-His215 anion, in turn, is protonated in a redox-process, which most probably includes bicarbonate, D1-Tyr246 (Fig. **2**, centre panel) and water as well as rearrangement of H-bonds network within the Q_B_ binding niche.

### 2.3. Additional PSII Plastoquinone Binding Sites

A third plastoquinone molecule (Q_C_) associated to PSIIcc was identified in the 2.9 Å resolution crystal structure of *T. elongatus,* and five of its isoprenoid units were successfully modelled into the PQ/PQH_2_ exchange channel I, between the TMH of PsbJ and Cyt.*b_559_* *α* and *β*-subunits [5]. In a subsequent 3.6 Å resolution crystallographic study of monomeric PSII core complex from *T. elongatus*, a trace of electron density was revealed in the same position but it was not possible to assign it to a specific molecule [6]. No electron density was detected in the putative Q_C_ binding side in themost detailed 3D structure at 1.9 Å resolution from 

*T. vulcanus* [8], and this binding spot was neither occupied by PQ nor by PSII herbicide terbutryn in *T. elongatus* PSII/terbutryn crystal complex resolved at 3.2 Å resolution [7]. The uncertainty of Q_C_ presence in the different structures has been ascribed to differences in adopted purification and crystallization procedures, and photosynthetic sample pre-treatment, to the low binding affinity of Q_C_, or to the lack of a well-defined binding pocket [5, 7]. In fact, neither H-bonds nor π–π interactions were observed for Q_C _stabilization and, most probably, the molecule binding is a result of the possible van der Waals interactions with the nearby lipid molecules, ChlD2, CarD2 and phytyl tail of Q_B_ [5, 23], Table **2**, PQ Head attractive interactions and (Fig. **2**). FMO calculations based on the 2.9 Å resolution structure pointed out that more than 90% of the Q_C_ interaction energy with the protein matrix and with PSII integral lipids and cofactors is due to its isoprenoid chain (Table **1**). In addition, the relative contribution of the interactions with the surrounding protein matrix to Q_C_ total binding energy is about 56%, that is much lower compared to that calculated for Q_A_ and Q_B_ [23] – about 100% and 82%, respectively (Table **1**). The high mobility of the Q_C_ molecule has been attributed to its peculiar interaction with the environment, which is characterized by the head group rimming predominantly by a belt of lipids and cofactors, and the tail fencing to proteins (Table **2**). The X-ray localization of a third PQ molecule in the 2.9 Å PSII structure supported earlier findings on the occurrence of additional quinones (different from Q_A_ and Q_B_), located in the PQ hydrophobic cavity close to the Cyt.*b_559_* [44, 45]. The physiological role of the Q_C_ is still under investigation. Guskov and coworkers [5] proposed three hypothetical mechanisms for PQ/PQH_2_ exchange, in which Q_C_ is alternating with Q_B_ or is the PQ molecule waiting to enter into the Q_B_-binging site, thus contributing to the rapid turnover of the PQH_2_ with a new PQ molecule from the membrane pool. Experimental evidences have suggested a strong involvement of Q_C_ in a PSII photoprotective electron pathway mediated by Cyt.*b_559_* [20-22]. It was hypothesized that Cyt.*b_559_* could act as a plastoquinol oxidase (oxygen reductase) or a superoxide oxidase/reductase enzymes; these functions have been extensively documented in other reviews [15, 16, 36]. A distinctive feature of this heme-containing protein is its redox heterogeneity. In PSII membrane fragments 75% of Cyt.*b_559_* is in its high potential form, 16% is in its intermediate potential form and 9% is in its low potential form; its redox properties and reactivity have been correlated to the occupancy of the Q_C_ site [22]. A biphasic pattern of Cyt.*b_559_* reduction by PQH_2_ has been observed in PSII membrane fragments containing predominantly oxidized Cyt.*b_559_*, and experimental results suggested that a PQ/PQH_2_ molecule bound to the Q_C_ site is responsible for the fast phase (with rate constant of 100 ms^−1^) of the Cyt.*b_559_* reduction [46]. The slow Cyt.*b_559_* reduction phase (with rate constant of 2.4 min^−1^) was attributed to formation of redox equilibrium between a set of Cyt.*b_559_* redox forms and “free” PQ/PQH_2_ from the membrane pool. The most likely mechanisms suggested by the authors for the Cyt.*b_559_* fast reduction requires: *i*) a location preferably encouraging one-electron redox-reaction; *ii*) a polar environment allowing accommodation of the PQ/SQ/PQH_2_ and occurrence of the so called L substances in anionic form (such as ADRY reagents, dinoseb, high concentration of DCMU, tetraphenylboron); and *iii*) a fine-tuned Cyt.*b_559_*/quinone interaction requiring well-defined quinone-protein interactions. However, the structure of the Q_C_ binding site described in the 2.9 Å resolution crystallographic study does not fulfil any of these requirements. The discrepancy between the structurally identified and experimentally suggested additional PSII PQ binding sites was found also in previous studies [20-22]. In order to distinguish between the Q_C_ site and the presumed polar quinone site of PSII that binds PQ/SQ/PQH2/L, the authors proposed to assign the latter as Q_D_ [46]. These experiments led to hypothesize the existence of two different PQ binding sites, Q_C_ and Q_D_, which host two different PQ molecules or one bound quinone that can occupy two different positions. 

## 3. DYNAMICS STUDIES IN PHOTOSYNTHESIS RESEARCH

### 3.1. Neutron Scattering Techniques

The determination of protein structure and the analysis of conformational changes are the key necessary to understand the biological function and the activity of a protein. The structural characterization of proteins, however, constitutes only part of the puzzle because their function requires a specific dynamic. The protein motions arise on a wide range of timescales, from femtoseconds for bonds vibrations to milliseconds or seconds, for more complex processes that require a structural rearrangement. The dynamics of proteins contributes to the thermodynamic stability of their functional states and plays an important role in various processes, such as allosteric processes, where the correlations between structural fluctuations are able to transmit information between distant sites of the protein. 

The study of dynamic processes at the microscopic level has been developed using a wide range of high resolution spectroscopies (Mössbauer spectroscopy, nuclear magnetic resonance (NMR), time-resolved fluorescence). We should add elastic, quasi-elastic and inelastic neutron scattering (ENS, QENS and INS, respectively) technique, which offers the advantage of covering length/distance (0.5-10 Å) and energy (0-200 meV) scales that correspond to thermal fluctuations and provide information on the geometry of the movements [47, 48]. The neutrons as a dynamics probe are highly sensitive to movements of hydrogen atoms, which are uniformly distributed within biological structures and represents nearly half of the atoms in them. On temporal and spatial scales accessible with neutron scattering, the hydrogen atoms trace the movements of the chemical groups, which they are linked to, such as amino acids side chains. In addition, it is also possible to mask part of the signal using specific deuteration, since deuterium has a weak scattering signal with respect to that of hydrogen [48]. The neutron scattering experiments, with their peculiar chance to exploit the isotopic substitution technique, permit to integrate and give complement to data obtained by numerical simulation and through specific molecular biology techniques. Moreover, they provide unique information useful for the characterization of the dynamical heterogeneity of biological macromolecules, from globular protein up to membrane complexes, in the temporal range 10-12 - 10-9 s.

The neutron scattering is an invaluable technique to tackle a plethora of important open questions in Biophysics such as the definition of correlations between structure, dynamics and functionality, the correlations between the interaction of water molecules with proteins and its role in the folding process and enzymatic activity, the observation of how the hydrogen bond network between solvent and biological macromolecules is affected and modified by selective genetic mutation, and hence the local flexibility and biological efficiency of the molecules [49-51]. 

### 3.2. Neutron Scattering in Photosynthetic Research

Photosynthetic research exploring the relationship between the function and the dynamics is a new field of interest. However, neutron scattering has been demonstrated to be a suitable tool to investigate protein dynamics in both complex multi-subunit photosynthetic systems and whole cells [52, 53]. 

A pioneer quasi-elastic neutron scattering study on the purple bacterium *Rhodobacter sphaeroides* RC proteins has demonstrated the effect of genetic point mutations for the overall protein dynamics [52]. The RC dynamics over a temperature range of 280 K was probed in a wild type and two non-functional mutants, carrying alanine residues on the place of Glu212 and Asp213 in the L-subunit. The Glu212 residue is highly conserved in bRC and is involved in the secondary ubiquinol protonation (see section 2.2). The crystallographic structure of the mutant proteins showed a local configuration of the active site more open than that of the wild type. One of the parameters indicative for the internal protein motions, the mean square displacement (<u^2^>), calculated for the two mutants resulted to be higher than for the wild type protein suggesting a higher flexibility [52]. These results correlated the enhanced flexibility of mutated RC proteins with a lack of functionality, thus suggesting the requirement of a rigid core for the accomplishment of protein function in a multi-protein complex, such as RC involved in highly tuned photosynthetic reactions. This ground-breaking experiment opened the way to the urgency to understand if the core rigidity is essential for the charge transfer process, and if this property is shared by all the photosynthetic systems, and how this information can be applied to the design of bio-sensors and organic semiconductors.

An original green microalga species from the *Chlorococcal* order, which was never studied for its high tolerance to radiations, has been isolated in the Institute Laue Langevin research reactor storage pool. The microorganism able to live and reproduce in extreme environments was investigated by Farhi and coworkers combining neutron scattering experiments of internal dynamics with results of radiation effects on the microalgae physiology, metabolism and genomics [53]. The microalgae were stressed with radiation doses up to 20 kGy (2 Mrad), and studied by NMR, looking for modification in the metabolism, and by ENS, looking for both dynamical flexibility and structural macromolecular changes in the cells. While the sugar metabolism was slightly reduced and only at high radiation dose, it was probed that the effect of a high irradiation on micro-algae does not lead to protein denaturation, but rather to reduction of macromolecules flexibility – as showed by the reduction of mean square displacement (<u^2^>) in (Fig. **2**) of reference [53]. 

Pieper and coworkers have performed exhaustive neutron scattering studies on protein dynamics of PSII membrane fragments and antenna complex LHCII as a function of the temperature and sample hydration level [54]. They showed that PSII membrane fragments undergo a hydration dependent ”dynamical transition” at about 240 K, underling the crucial role of hydration water for PSII flexibility. Above this temperature, PSII membrane fragments exhibit structural flexibility due to the presence of fast (picosecond) internal protein motions, while there is only harmonic vibrational dynamics at lower temperatures. It was then speculated that diffusive protein dynamics are indispensable for enabling Q_A_ reoxidation by Q_B_ at temperatures above 240 K, which explains the strong dependence of this electron transfer step on temperature and hydration level of the sample.

A newly developed technique of time-resolved QENS offers the advantage of simultaneous monitoring of protein internal dynamics and functional activity in specific sample functional states. This method was firstly applied for dynamics-function correlation in bacteriorhodopsin, a membrane embedded protein that functions as a light-driven proton pump in *Halobacterium salinarum*, and lately for the more complex PSII membrane fragments with inhibited Q_A_ to Q_B_ electron transfer [54, 55]. At physiological temperatures the calculated mean-square displacement of the hydrogen atoms, characterizing the flexibility of the system, revealed a significantly increased flexibility between 310 and 320 K [56]. The authors claimed that this transition is evidently correlated with the detachment of OEC from the membranes, as suggested by its absence in samples in which OEC has been removed prior to the QENS experiments. The transition is not accompanied by significant changes in the molecular organization of the pigment protein complexes, but the role of monomerization of PSII dimers cannot be ruled out.

Lately, preliminary results on intracellular water collective dynamics investigation on *Chlamydomonas* cells, carrying both native and mutated D1 RC proteins, have been also addressed. The authors proposed a comparison between fully hydrated and partially de-hydrated deuterated whole cells. It was observed a distinct sound propagation speed between the two hydration levels suggesting a more rigid structure of hydration water than intracellular water [57]. 

## 4. COMPUTATION ANALYSES FOR MODIFICATION OF Q_B_ BINDING POCKET AMINO ACID COMPOSITION: FUNDAMENTAL RESEARCH AND APPLICATIONS

In the last decades, several mutational studies on D1 protein were conducted to determine the effects induced by aminoacidic substitutions located in the stromal loop of the D1 protein. Mutations resulted in an impairment of the electron transfer between Q_A_ and Q_B_ leading to the identification of residues often involved in modulation of PSII photoinhibition susceptibility and/or herbicide specificity [58-62]. Computational studies on PSII D1 protein aimed at rational modification of the Q_B_ binding pocket have been scarce partly because a high resolution three-dimensional structure of PSII has been available only relatively recently and partly due to the difficulties in generating site-specific mutants of this protein.

The first structure of PSII from *T. elongatus* has been published only in 2004 and at a resolution of only 3.5 Å [PDB code 1S5L,^ 3^]. The same year a 3.2 Å resolution structure has been published independently [PDB code 1W5C,^ 63^] and refined the following year at 3.0 Å resolution [PDB code 2AXT,^ 4^]. However, this last structure had still an insufficient resolution to uncover all the atomic details of this macromolecular proteins-cofactor complex such as the precise architecture of the OEC. Nonetheless, the Loll *et al.* crystal structure was sufficiently detailed to allow the first computational studies on the Q_B_ binding pocket as well as homology modelling of PSIIs from other organisms. The first of such studies was probably that of Loll and coworkers [64] who modelled the three different copies of *T. elongatus* D1 protein (PsbA1-A3) within the 3.0 Å resolution crystal structure of PSII, revealing that most of the D1 amino acid variants were located in the direct vicinity of redox-active cofactors of the electron transfer chain. However, proper computational studies aimed at selecting amino acid variants within the Q_B_ binding pocket able to modify the recognition properties of the pocket itself appeared only the following year [65]. In this study, homology modelling of the *Chlamydomonas reinhardtii* D1 protein was carried out based on the *T. elongatus* crystal structure, followed by *in silico* mutagenesis and energy minimization analyses to identify functional amino acids in the D1 protein interacting with herbicides. Following the results of theoretical calculations, three site directed mutants were produced by site-directed mutagenesis and characterized by fluorescence analysis. This study demonstrated that D1 mutants S268C and S264K could be used in the development of biomediators suitable for the selective detection of triazine and urea classes of herbicides, respectively. The study was further refined the same year undertaking an *in silico* study of the *C. reinhardtii* D1-D2 proteins with the aim of designing mutants with increased affinity for atrazine [66]. The final outcome of this work was the design and selection of D1 mutants hypothetically able to increase atrazine binding affinity by orders of magnitude, representing a useful theoretical guide for the development of high affinity atrazine biosensors.

**Fig. (3):**
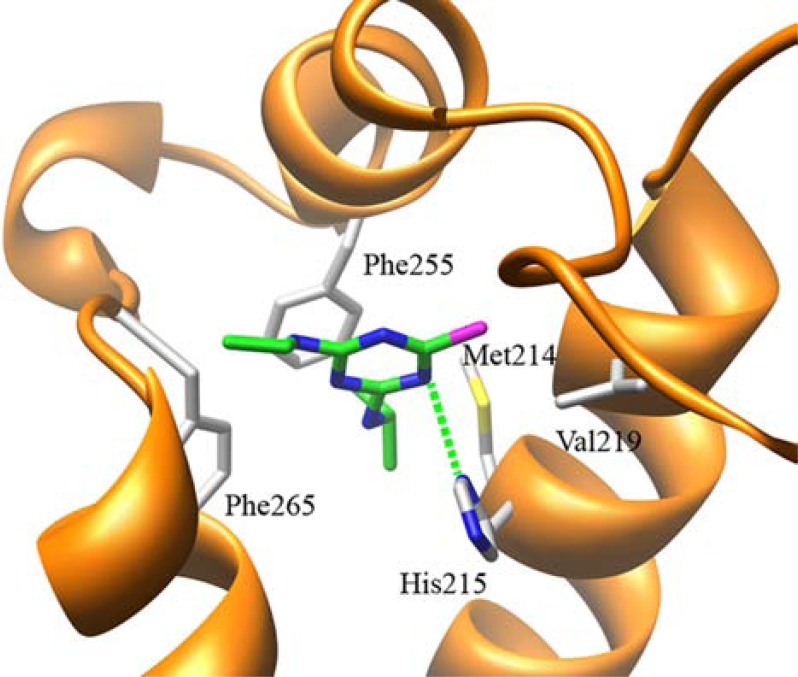
Schematic view of the interactions of atrazine within the PSII QB binding pocket predicted through a 10 ns MD simulations run [66]. For clarity, only the D1 backbone (orange ribbon) and residues mentioned in the text are shown. The dashed green line highlights the hydrogen bond between atrazine and His215. Atrazine carbon atoms are in green, nitrogen atoms are in blue, sulphur atom is in yellow and the chloride atom is in magenta.

With the increase in computing power, it has become feasible to adopt more sophisticated structural bioinformatics approaches, such as molecular dynamics (MD) simulations, for the study of the recognition properties of the Q_B_ binding pocket toward natural and exogenous ligands. The first all atoms MD simulations of the complete *T. elongatus* PSII structure (including all cofactors) embedded in a lipid membrane was published in 2011 [67]. The aim of this work was that of uncovering the fine details of atrazine molecular interactions within the Q_B_ binding pocket using a full atomistic (and thus realistic) description of PSIIcc. Atrazine was first docked in the vicinity of the Q_B_ binding pocket and then let free to diffuse during a 10 ns MD simulations trajectory. During the simulations atrazine bound deeper into the Q_B_ pocket establishing hydrophobic interactions with PSII D1-Phe255 phenyl ring and D1-Met214 side chain, and a single strong hydrogen bond with D1-His215 (Fig. **3**). On the other hand, no binding partner appeared to stabilize other groups of the atrazine molecule such as the chloride atom (bound in an energetically unfavourable position in the vicinity of the aliphatic residue D1-Val219) and the N4 and N5 protons for which no hydrogen bonding partner was observed. The conclusions of this study were that rational design of site-directed mutants was likely to increase atrazine affinity for the Q_B_ binding pocket. In particular, it was suggested that, among others, mutation of D1-Phe265 residue with a polar hydrogen bond acceptor residue could stabilize the atrazine N5 proton and atrazine binding within the pocket.

## CONCLUSIONS AND PERSPECTIVES

The advances in the crystallographic characterization of PSIIcc have broaden the understanding of PSII function and organization allowing to identify the atomic interactions taking place between the RC protein matrix, electron transport cofactors and thylakoid membrane lipids. Unravelling finer molecular details and mechanisms of the PSII structure, function and dynamics is mandatory to support novel biotechnological applications exploiting its unique properties of charge separation, electron transfer and water splitting. Specifically, it is possible to exploit PSII enriched particles or their active subcomponent to develop receptor elements suitable to be exploited in photosynthesis-based biosensors for the detection of environmental pollutants. The latter, in fact, can interact with the PSII donor side interrupting the light-induced electron transfer, generating a measurable opto-electronically detectable signal. In this context, insights into atomic interactions and dynamics of polypeptides and co-factors provided by both molecular dynamics or docking studies will help to design novel bio-recognition elements having improved selectivity and sensitivity towards different classes of compounds. Studies exploring the relationship between the function and the dynamics of the photosynthetic complex systems are still in their nascent stages, along with MD simulations. However, these techniques could offer a real possibility of investigating the dynamics associated with a molecule’s biological function and complement the progress of this technology. Besides, the extraordinary properties of water-splitting could be used to design bio-inspired device for bio-energy production, while the charge separation features could be exploited to design molecular photovoltaics. 

## Figures and Tables

**Table 1.** Distribution of interaction energy of the PSII plastoquinone within Q_A_, Q_B_ and Q_C_ binding niche, according to Hasagawa and Noguchi [23]. The relative contribution of the protein matrix, and lipids and cofactors, respectively, are calculated as a percentage of the total interaction energy of the corresponding PQ molecule. The head/tail distribution of the total PQ interaction energy, as well as with the protein matrix and lipids and cofactors are calculated in a similar manner. The relative contribution of the PQs phytyl tail and head group for the total molecule interactions are also presented. Correlation between the increased PQ mobility (Q_A_>Q_B_>Q_C_) and reduction in the interaction with surrounding protein matrix is underlined.

Factors Contributing to PQs Interactions	Interaction Energy Distribution, %		
PQ at QA Binding Niche	PQ at QB Binding Niche	PQ at QC Binding Niche
Protein matrix PQs interactions	100.0	82.0	56.0
PQs head contribution	38.0	44.9	8.8
PQs tail contribution	62.0	56.1	91.2
Lipids and cofactors PQs interactions	None	18.0	44.0
PQs head contribution	-	0.0a	10.0
PQs tail contribution	-	100.0	90.0
Relative head contribution to total PQs interaction energy	38.0	35.6	44.6
Relative tail contribution to total PQs interaction energy	62.0	64.4	55.4

a The repulsive QB interactions with lipids and cofactors counteract to the attractive one.

**Table 2.** Factors involved in PSII plastoquinone interactions within Q_A_, Q_B_ and Q_C_ binding niche, summarized from Hasagawa and Noguchi [23]. Amino acid residues providing attractive or repulsive effects with |∆E’|≥2.0 kcal/mol and lipids and cofactors providing attractive or repulsive effects with |∆E’|≥0.1 kcal/mol are presented. Amino acids, lipids and cofactors are ordered following the decrease of the interaction energy with the corresponding PQ molecule.

Factors Involved in PQs Interactions	PQ at QA Binding Niche	PQ at QB Binding Niche	PQ at QC Binding Niche a
Protein Matrix			
Head attractive interactions	D2-Phe261, -Thr217, D2-Trp253, -His214, D2-Met246, -Ser262	D1-Phe255, -Phe265, D1-Val219, -Leu218, D1-His215	None
Tail attractive interactions	D2-Gly200, -Gly203, D1-Phe52, PsbL-Leu30, PsbT-Phe10, PsbL-Leu27, D2-Phe270, D1-Ile77	D2-Phe38, -Tyr42, D1-Phe211, PsbF-Leu26, -Thr25, D2-Arg26, -Arg128	PsbJ-Gly20, -Val21, PsbJ-Gly18, -Ala17, PsbJ-Met19, -Ile24, PsbF-Thr30, -Ile31, PsbE-Phe31
Repulsive interactions	Head: D2-Gly215, D2-Arg251 Tail: D2-Leu279, D2-Val204, -Val274	Head: D1-Gly253, D1-Asn266 Tail: D2-Leu43, PsbF-Val23, -Ala22, D1-Leu271, D2-Asp25 PsbE-Glu7	Head: PsbE Tail: PsbT
Lipids and Cofactorsb			
Head attractive interactions	-	DGDG6	MGDG18, ChlD2, MGDG7, CarD2, DGDG6, SQDG4
Tail attractive interactions	-	ChlD2, MGDG18, SQDG4, DGDG6, MGDG7	CarD2, MGDG18, DGDG6
Repulsive interactions	-	Head: MGDG18, ChlD2	Tail: MGDG7, CarD2,

a For Q_C_ all PSII amino acid residues providing attractive effects with |∆E’|≥1.0 kcal/mol are presented.

b The abbreviations for lipids and molecular cofactors are same as define in Section 1 and the List of Abbreviations.

## LIST OF ABBREVIATIONS

ADRY: = Acceleration of the deactivation reactions of system Y
bRC: = Bacterial reaction centre
Car: = Carotenoids
Chl: = Chlorophyll
CP43: = PSIIcc chlorophyll apoprotein encoded by *psb*C gene
CP47: = PSIIcc chlorophyll apoprotein encoded by *psb*B gene
Cyt.b_*559*_: = Cytochrome *b_559_*
DGDG: = Digalactosyldiacylglycerol
ENS: = Elastic Neutron Scattering
FMO: = Fragment Molecular Orbital
INS: = Inelastic neutron scattering
L substances: = Chemical compounds that induce negative shift in the midpoint potential of high potential form of Cyt.*b_559_*
LHCII: = Light Harvesting Complex II
MD: = Molecular Dynamics
MGDG: = Monogalactosyldiacylglycerol
NMR: = Nuclear Magnetic Resonance
OEC: = Oxygen Evolving Complex
PDB: = Protein Data Base
PG: = Phosphatidylglycerol
RC: = Reaction Centre
Pheo: = Pheophytin
PQH_2_: = Plastoquinol
PQs: = Plastoquinones
PsbE and PsbF PSIIcc proteins: = Corresponding to *α-* and *β-*subunit of cytochrome *b_559_*
PSI: = Photosystem I
PSII: = Photosystem II
PSIIcc: = PSII core complex
Q_A_: = PSII primary quinone electron acceptor
Q_B_: = PSII secondary quinone electron acceptor
Q_C _and Q_D_: = PQ molecules supposed to be bound to PSIIcc
QENS: = Quasi-elastic neutron scattering
SQ: = Semiquinones
SQDG: = Sulfoquinovosyl-diacylglycerol
terbutryn: = 2-*tert*-Butylamino-4-ethylamino-6-methylthio-1,3,5-triazin
TMH: = Trans-membrane helices.
